# Effect of Laser Remelting on Cladding Layer of Inconel 718 Superalloy Formed by Laser Metal Deposition

**DOI:** 10.3390/ma13214927

**Published:** 2020-11-02

**Authors:** Bo Xin, Jiangyu Ren, Xiaoqi Wang, Lida Zhu, Yadong Gong

**Affiliations:** School of Mechanical Engineering and Automation, Northeastern University, Shenyang 110819, China; jiangdou361@163.com (J.R.); 2070224@stu.neu.edu.cn (X.W.); neulidazhu@163.com (L.Z.); gongyd@mail.neu.edu.cn (Y.G.)

**Keywords:** laser remelting (LR), laves phases, microstructure, laser scanning speed

## Abstract

The brittle phase (Laves) of Inconel 718 parts formed by laser metal deposition (LMD) represents a bottleneck of the engineering applications. In order to investigate effectiveness of laser remelting (LR) technology on suppressing the formation of Laves phase, different laser scanning speeds of the LR process were adopted to build and remelt the single-pass cladding layers. The evolution of phase composition, microstructural morphology, and hardness of the LMD and LMD + LR specimens were analyzed. The experimental results show that different laser scanning speeds can obviously change the microstructural evolutions, Laves phase, and hardness. A low laser scanning speed (360 mm/min) made columnar dendrite uninterruptedly grow from the bottom to the top of the cladding layer. A high laser scanning speed (1320 mm/min) has a significant effect on refining Laves phase and reducing Nb segregation. When the laser scanning speed of LR process is equal to that of LMD, the cladding layers can be completely remelted and the content of Laves phase of the LMD + LR layer is 22.4% lower than that of the LMD layer. As the laser scanning speed increases from 360 to 1320 mm/min, the mean primary dendrite arm spacing (PDAS) values of the remelting area decrease from 6.35 to 3.28 μm gradually. In addition, the low content of Laves phase and porosity contribute to the growth of average hardness. However, the laser scanning speed has a little effect on the average hardness and the maximum average hardness difference of the LMD and LMD + LR layers is only 12.4 HV.

## 1. Introduction

Laser metal deposition (LMD) process is an additive manufacturing technology that is useful for the production of large or high-valued metal components in a short production cycle [1]. By utilizing a high-energy laser, a molten pool is formed by melting the alloyed spherical powder ejected from several nozzles, and then rapidly solidifies to deposit cladding layers. LMD has been successfully employed to form many types of metallic materials and applied in aeronautics, astronautics, mould, etc. Recently, LMD of Inconel 718 Ni-based superalloy has attracted much attention because of the benefits of excellent high-temperature creep resistance and good metallurgical bonding with less heat-affected zone when compared to traditional metal joining processes [2]. The Inconel 718 superalloy has excellent heat resistant performance (650–1000 °C) and stress-corrosion resistance [3,4,5]. Thus, the Inconel 718 structures have been widely used in aviation and aerospace, especially for the turbine blades [6,7]. However, previous research results show that the brittle phase (Laves) inevitably appears during the laser cladding process of Inconel 718 and some strengthening phases are difficult to precipitate in the forming structure [8]. Meanwhile, due to the rapid cooling and solidification of the process of direct laser deposition, internal defects such as pores, non-fusion, and cracks are easily produced in the cladding layers [9]. These defects can greatly weaken the mechanical properties of Inconel 718 structures and restrict its wide application.

To address these issues, extensive research has been launched in recent years to decrease the content of Laves phase. Some research focuses on optimizing the LMD parameters such as laser scanning speed and laser beam power to reduce the energy input [10,11]. However, too low energy input will cause more cracks and pores [12]. Xiao et al. [13] found that the quasi-continuous-wave (QCW) laser tends to produce fine equiaxed dendrites, less Nb segregations and finer discrete Laves phase than the continuous-wave (CW) laser.

In addition, some auxiliary process was applied in the LMD to control the Laves phase. For example, Zhang et al. [14] found that the content of Laves phase in the cladding layer decreased from 10.25 to 3.5 vol.% when the substrate was placed in liquid nitrogen. Li et al. [15] indicated that the solution and double aging treatment made the Laves phases gradually dissolve and many strengthening phases precipitated at the dendrite boundaries with the solution temperature rising. Besides these methods, laser remelting (LR) had been proved to reduce the porosity and improve the surface performance of the LMD structures [16]. For instance, Lu et al. [17] found that LR treatment had a significant influence on surface topography of K423A nickel-base superalloy and appropriate LR parameters can improve the surface properties of material. Yasa et al. [18] indicated LR treatment can improve the density (up to 98%) and hardness of the remelted zone when the energy density is sufficient. Wei et al. [19] optimized the number of remelting cycles to improve the residual stress of the LMD structure.

In addition to the above methods based on LR treatment, Mo et al. [20] indicated that vanadium (V) may effectively prevent the precipitation of Laves phase (volume fraction reduced from 2.3% to 0.4%). Micro-alloying of V can be very helpful in ingots homogenization annealing of Inconel 718 alloy in industry production.

Based on the above analysis, this research fully recognizes the contributions of the existing literatures. However, to the best of the authors’ knowledge, effects of laser remelting treatment on phase composition of Inconel 718 superalloy structures by laser metal deposition still need more investigations. Thus, in this study, hybrid LMD and LR processes with different laser scanning speeds were adopted to form the single-pass cladding layers in order to analyze the evolution of microstructural morphology, phase composition and hardness of Inconel 718 superalloy. The experimental results verified the feasibility of the LR treatment.

## 2. Experimental Condition and Procedure

### 2.1. Experimental Condition and Material

**Figure 1:**
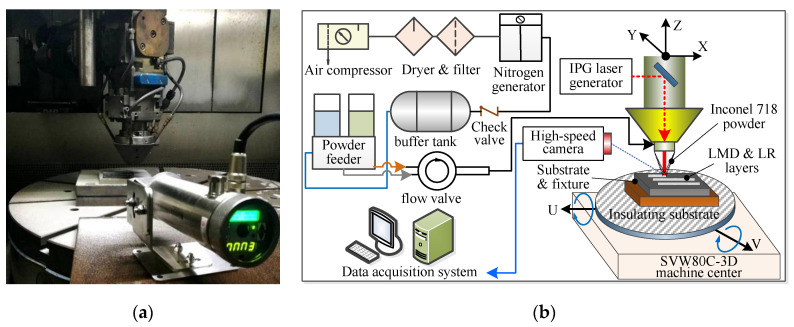
(**a**) SVW80C-3D hybrid additive and subtractive machine center; (**b**) principle of LMD and LR process in the SVW80C-3D center.

In this paper, the LMD and LMD + LR experiments of Inconel 718 superalloy were conducted in a SVW80C-3D (Hybridwise Technology Co. Ltd., Dalian, China) hybrid additive and subtractive machining center, which is primarily composed of YLS-2000 laser generator (IPG Photonics Corporation, Oxford, MS, USA), RC-PGF-D-2 powder feeder (Zhongke Raycham Laser Technology Co., Ltd., Nanjing, China), ET-80 air compressor (Jaguar Mechanical and Electrical Equipment Co., Ltd., Shenyang, China), DM-1.5H nitrogen generator (Demiao Technology Co. Ltd., Shijiazhuang, China), Heidenhain operating system (TNC640, Heidenhain, Berlin, Germany) and data acquisition system. The laser scanning speed can be adjusted by controlling the movement speed of the 3-axis laser cladding head, as shown in Figure 1a. Nitrogen gas is selected as protecting and carrier gas to deliver the powder to the substrate. In the LR process, the powder feeder is turned off to stop delivering powder when the laser generator works. Figure 1b illustrates the principle of LMD + LR processes.

A nickel-based alloyed Inconel 718 powder with the spherical shape was selected as laser deposition and remelting material in this research. The distribution of particle size is in a range of 53–150 μm. The chemical composition of the Inconel 718 powder is given in Table 1. In order to achieve a good metallurgical bonding and reduce cracks in the interface between the substrate and Inconel 718 cladding layers, we chose the forged Inconel 718 superalloy as the substrate with the size of 160 × 100 × 10 mm^3^. Moreover, before the LMD/LR process, the substrate surface was well cleaned.

### 2.2. Experimental Parameter and Procedure

**Figure 2:**
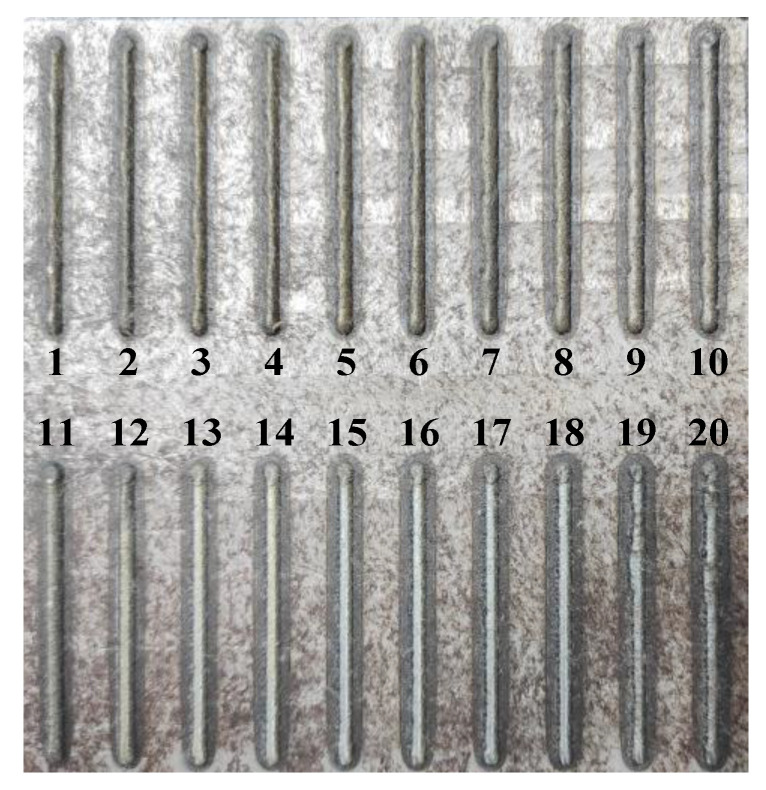
The formed LMD and LMD + LR single-pass layers in the experiments.

The previous studies have shown that laser power and laser scanning speed play an important role in the performance of LMD process [21]. In this research, the laser scanning speed $v_{\mathrm{LR}}$ of the LR process was selected as a single variable in a range of 360 mm/min to 1320 mm/min and the increment $\Delta v_{\mathrm{LR}}$ was 480 mm/min. Other constant parameters of the LMD process were set as follows: laser cladding power $P_{\mathrm{LMD}}$ was 1000 W; powder feed rate was 13.5 g/min; laser scanning speed $v_{\mathrm{LMD}}$ was 360 mm/min. Moreover, the distance from the focus of laser beam to molten pool (defocusing distance, $L_{D}$) was 13.5 mm. As each cladding layer had been deposited and solidified, LR treatment was followed and the remelting direction keeps constant with the LMD process. The laser remelting power $P_{\mathrm{LR}}$ was 1000 W. The formed LMD and LMD + LR samples in the experiments are shown in Figure 2, including 11 LMD layers (No. 1–11) with the same technological parameters and 9 LMD + LR layers (No. 12–20) which were divided into three groups by using the laser scanning speeds of 360 mm/min (No. 12–14), 840 mm/min (No. 15–17), and 1320 mm/min (No. 18–20), respectively.

In the experiments, the measuring items including hardness, dendrite morphology, phase structure of precipitation, especially Laves phase, and the chemical composition of the precipitations were implemented in the different regions of single-pass cladding and remelting layers. The results were compared with corresponding un-remelted layers. The metallographic cross sections of the LMD and LR specimens were obtained through cutting, grinding, polishing, and etching (40 mL HCl + 40 mL C_2_H_6_O + 2 g CuCl_2_). A high-speed camera (5KF20, FuHuang AgileDevice (Revealer), Hefei, China) was used to visualize the LMD and LR processes and take instantaneous pictures of the molten pool under more than 5000 frames per second (FPS). The OLS4100 3D microscope (Olympus Corporation, Tokyo, Japan) and Zeiss’s ULTRA PLUS (Carl Zeiss AG, Oberkochen, Germany) scanning electron microscope (SEM) were used to observe the dendrite morphology and precipitated phase respectively. The chemical composition of the phase in the molten was characterized by the energy spectrometer with SEM. The micro-hardness measurement was conducted on the HVS-1000M Vickers hardness tester (Ledi Instruments Co., Ltd., Ningbo, China) (test pressure 1 kg, the loading time and duration were 10 s and 3 s respectively).

## 3. Results and Discussion

**Figure 3:**
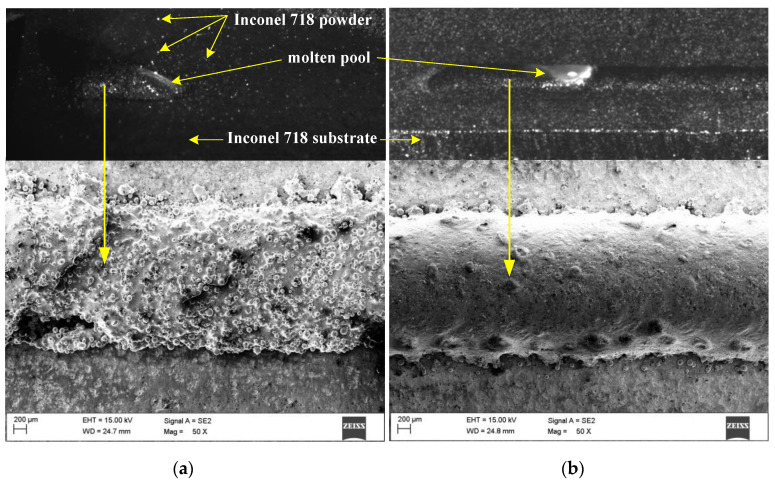
Surface morphologies of the molten pool and single-pass cladding layers: (**a**) LMD, (**b**) LMD + LR.

In the first experiment, the surface morphologies of the molten pool and single-pass cladding layers formed by LMD and LMD + LR processes were obtained by the high-speed camera and 3D microscope. It is evident from Figure 3 that the surface smoothness of the single-pass LMD layer was significantly improved by the LR process. During the LMD process, a mass of Inconel 718 powder was ejected from the nozzles and then was reflected by the substrate surface. Thus, much powder adheres to the top surface of cladding layer along the laser scanning track leading to the uneven surfaces and dents. After LR treatment, the adherent powder was remelted and flowed into the molten pool, which contributes to a better surface smoothness of the remelted layer.

### 3.1. Microstructure

**Figure 4:**
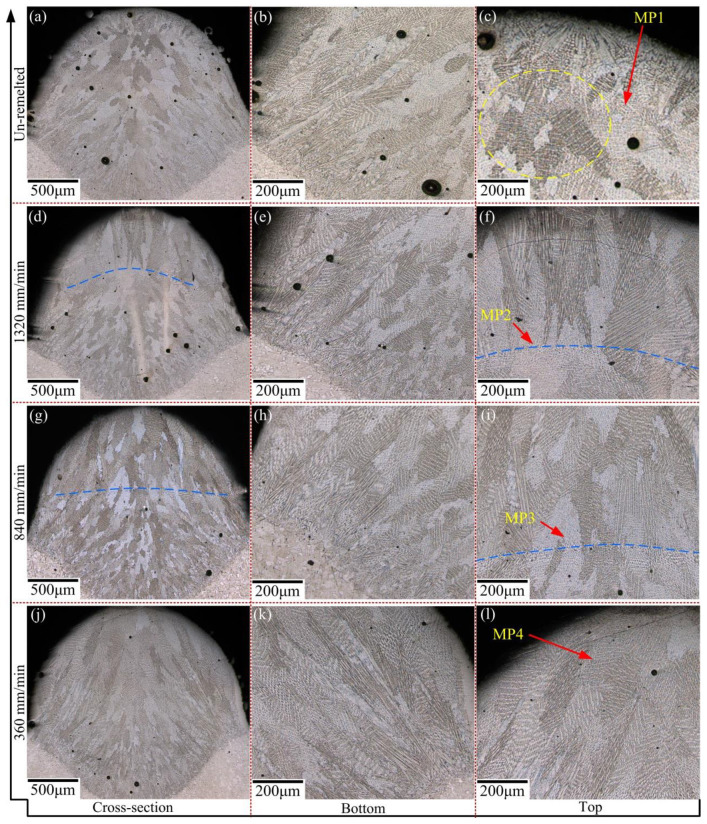
Dendrite morphologies of the cross section of the single-pass layers. (**a**–**c**) LMD; (**d**–**l**) LMD + LR: $v_{\mathrm{LR}}=$1320, 840 and 360 mm/min respectively.

Effect of laser scanning speed of LR process on the dendrite morphology was studied in this subsection. Figure 4 displays the cross sections of several single-pass specimens. For the LMD layers in Figure 4a–c, columnar dendrites grow along the bottom boundary and then transform to equiaxial dendrites in the middle and top of the cladding layer. As shown in the yellow dotted oval in Figure 4c, around the top of the layer, there exists a chaotic area where the equiaxial dendrites differ with each other in size and shape. Because fast heat dissipation through the substrate occurs at the interface between the cladding layer and the substrate, a high temperature gradient contributes to the formation of columnar crystals. As the distance from the substrate increases, thermal radiation and convection with air play a major role in heat dissipation. The gradual reduction of the temperature gradient is the main reason for the formation of equiaxial dendrites. Meanwhile the change of heat dissipation mode inevitably makes more impurities element in the air mix into the molten pool, which is beneficial for gathering particles of heterogeneous nucleation. So, an unordered crystallization of the equiaxial dendrites forms in the top the layer.

Based on Figure 4d–l, as for the LMD + LR layers, the LR process not only eliminates the top chaotic area, but also promotes the growth of columnar dendrite in the middle-upper parts of the layer. This is mainly attributed to the metal flow in the molten pool, which would shake and break the existing coarse dendrites and increase the nucleation particles. The Gaussian heat source adopted in the LR process increases the fluidity of the molten pool. During the LR process, the new energy input makes the molten pool form again. A large number of nucleated particles generate and the dendrites are obviously refined. Figure 4d,g show that the depth of remelted boundary (blue dotted line) is related to the laser scanning speed. As the laser scanning speed is relatively fast (such as 840 and 1320 mm/min), inadequate remelting energy leads to the forming of a remelted boundary. Above the remelted boundary in Figure 4f,i, the remelted zone consists of plenty of columnar dendrites, owning to the heat dissipation from the top surface of pre-deposition layer. During the LR process, more columnar dendrites are inclined to grow along the pre-deposition layer. Stronger solidification structure and more uniform grain size conduce to a better metallurgical bonding between the neighboring layers. When the LR and LMD processes were kept the same scanning speed ($v_{\mathrm{LR}}=v_{\mathrm{LMD}}=$ 360 mm/min), the columnar dendrites continuously grow from bottom to top in the complete remelted layer. Moreover, the equiaxial dendrite above the remelted boundary (MP2 and MP3) is finer than that of below the remelting boundary.

In addition, Figure 4 also indicates that the high energy density absorbed in the molten pool can suppress pores’ production. The number of pores in the cladding layer obviously decreases with a lower laser scanning speed. The leading factor is that LR process not only prolongs the wetting time of the molten Inconel 718 superalloy but also accelerates the internal flow of molten pool. Thus, more formed gas bubbles will escape from pores, leading to a lower porosity.

### 3.2. Precipitation

**Figure 5:**
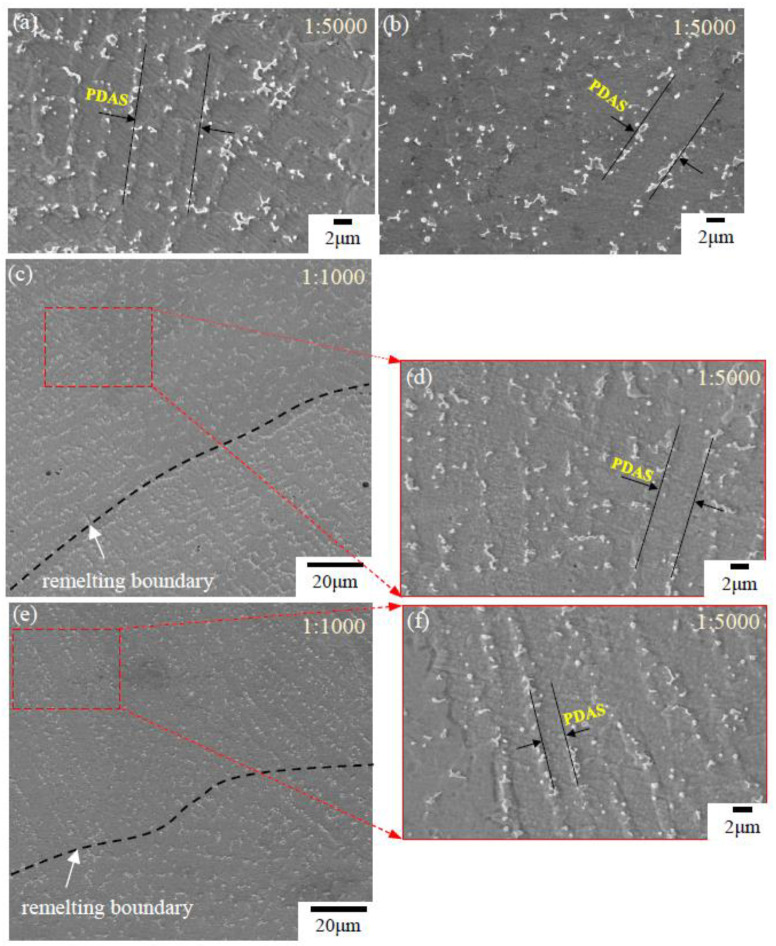
Microstructure morphologies and measured primary dendrite arm spacing (PDAS) on the cross sections of the single-pass layers. (**a**) MP1 in the LMD layer ($v_{\mathrm{LMD}}=$ 360 mm/min); (**b**) MP4 in the LMD + LR layer ($v_{\mathrm{LR}}=$ 360 mm/min); (**c**,**d**) MP3 in the LMD + LR layer ($v_{\mathrm{LR}}=$ 840 mm/min); (**e**,**f**) MP2 in the LMD + LR layer ($v_{\mathrm{LR}}=$ 1320 mm/min).

Figure 5 depicts a SEM analysis of precipitation structure of the LMD and LMD + LR specimens under different laser scanning speeds. Several measuring points on the cross sections of the single-pass layers (MP1-MP4 in Figure 4) were selected to observe the precipitation structure. Concretely, Figure 5a shows the larger versions (1:5000) of MP1 located in the top region of the LMD layer. Figure 5b shows the larger versions (1:5000) of MP4 located in the top region of the LMD + LR layer as $v_{\mathrm{LR}}=$ 360 mm/min. Figure 5c–f indicate the larger versions (1:1000 and 1:5000) of MP3 and MP2 located around the remelting boundary of the LMD + LR layers as $v_{\mathrm{LR}}=$ 840, 1320mm/min respectively. Based on Figure 5a, a large amount of white precipitates and gray matrix can be seen in the LMD layer. The white precipitates exhibit different morphologies, mainly occupied by continuous strip and irregular granule. The relatively fine and globular precipitation particles are mainly distributed around the mass precipitates.

**Figure 6:**
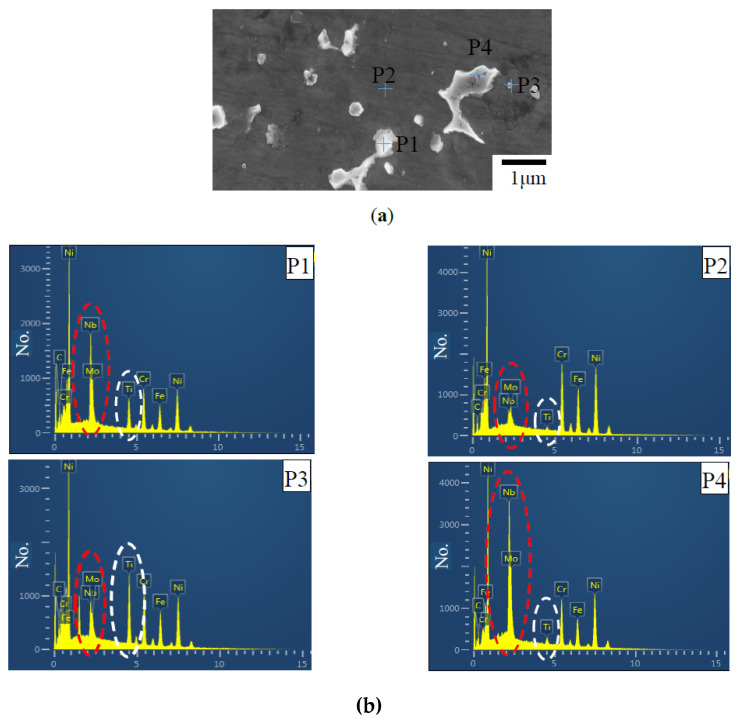
Comparison of different precipitates in the LMD + LR layer ($v_{\mathrm{LR}}=$ 360 mm/min). (**a**) Microstructure of different precipitates; (**b**) Energy spectrum analysis of different precipitates.

Furthermore, energy spectra of different precipitates (P1, P3, and P4) and gray matrix (P2) in the LMD + LR layer ($v_{\mathrm{LR}}=$ 360 mm/min) were measured and analyzed. Figure 6a displays the position of each probe point. Figure 6b and Table 2 show the elemental analysis results of different precipitates. The contents of element Nb and Mo in P1 are 19.04% and 7.75%, 6.2 times and 1.6 times higher than that in the gray matrix P2, 2.66% and 2.96%. Because of the enrichment of Nb and Mo, it can be judged that the Laves phase is mainly composed of the strip and irregular shaped precipitates such as P1 and P4. Based on the elemental result at P3, the globular precipitates are rich in C (9.07%), Ti (17.97%) and Nb (14.52%), especially the content of Ti reaches 25 times higher than that in the gray matrix P2, 0.69%. Thus, the globular phase can be identified as the MC carbides. According to the literature [22], the LMD process of Inconel 718 powders belongs to a non-equilibrium solidification and the microstructural evolution is L → L + γ → L + NbC/γ → L + Laves/γ. Nb is continuously ejected from the solid phase during the formation of dendrites because the solubility of Nb in solid phase is lower than that in liquid phase. As the solidification continues, the content of Nb increases obviously with the constant reduction of liquid phase in the molten pool. When the temperature of liquid phase reaches the eutectic point, the liquid phase transforms into Laves phase and γ phase. The white Laves phase is precipitated at the interdendritic regions. The edges of dendrites are visible due to the changing distribution of these bright phases. So, the morphology of the precipitates depends on the dendrites. Specifically, the strip-like Laves phase appears near the columnar dendrites and the network-like Laves phase appears near the equiaxed dendrites.

According to Table 2, it can also be found that the content of Ti in the white precipitates at P4 near the MC carbides is 1.46%, less than that of P1, 11.58%. In the solidification process, a lower solubility of Ti in solid phase makes much Ti continuously discharged into the liquid phase. Literature [23] proves that the density of Ti will increase in the liquid phase. Because the content of Ti in liquid phase is higher than Nb, a higher cooling rate would result in the first precipitation of the MC carbides.

**Figure 7:**
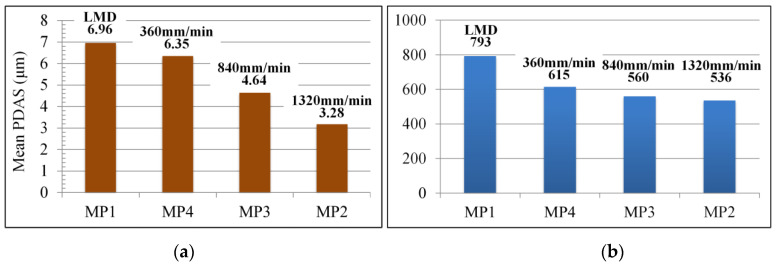
(**a**) Mean PDAS values of Laves phase in MP1–MP4; (**b**) The amount of Laves phase in MP1–MP4.

**Figure 8:**
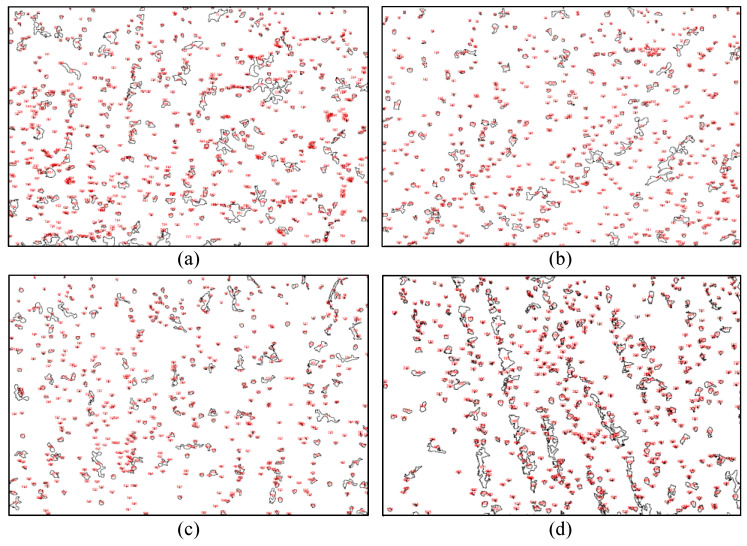
The marked Laves phase of the SEM images in Figure 5. (**a**) LMD layer ($v_{\mathrm{LMD}}=$ 360 mm/min); (**b**–**d**) LMD + LR layers ($v_{\mathrm{LR}}=$ 360, 840, and 1320 mm/min).

As for the primary dendrite arm spacing (PDAS), based on the SEM images of the precipitation structure in Figure 5, the PDAS was measured in 5 times at various positions on the SEM images for each laser scanning speed in order to obtain the mean PDAS values of Laves phase in MP1-MP4. As shown in Figure 7a, the mean PDAS values decrease from 6.35 to 3.28 μm gradually as $v_{\mathrm{LR}}$ increases from 360 to 1320 mm/min. Through comparison and analysis of the segregation phase content of the single columnar dendrite, it can be seen that a low laser scanning speed of LR process facilitates the precipitation of the Laves phase. To acquire the amount of Laves phase accurately, we adopted the image processing software ImageJ to analyze the images of the microstructure morphology (MP1–MP4) in Figure 4. A RenyiEntropy color thresholding method was used to mark the Laves phase and then the function of “Analyze Particles” was used to count the amount of Laves phase per unit area of 3.6 × 10^−3^ mm^2^. The results are shown in 7b and 8, which demonstrate that the amount of Laves phase is strongly influenced by the laser scanning speed. More specifically, based on Figure 7b and Figure 8a,b, the amount of Laves phase per unit area in MP4 of the LMD + LR layer is 615, 22.4% lower than that of the LMD layer, 793 as $v_{\mathrm{LMD}}=v_{\mathrm{LR}}=$ 360 mm/min. The Laves phase presents divergent distribution of dendrites because the energy density of the LR process is higher than that of LMD process with the same laser scanning speed and laser power. For the LMD process, the laser beam is always partially sheltered by the ejected powder, thus the energy density is lower than that of LR process. In the remelted layer, the concentration of Nb was restrained by large thermal gradient (rapid heating and cooling of molten pool) and more Nb was trapped in the gray matrix. Meanwhile, the flow of molten pool makes it more uniform in distribution of Nb, Mo, and Ti, which blocks the formation of Laves phase.

Moreover, as the laser scanning speed increases from 360 to 1320 mm/min, the amount of Laves phase in the remelting layer further decreases because a low scanning speed promotes the growth of dendrite, which contributes to the segregation of element Nb. When the laser power and scanning speed of LR process are equal to the LMD process ($v_{\mathrm{LR}}=v_{\mathrm{LMD}}=$ 360 mm/min), the cladding layer can be completely remelted and more columnar dendrites are found throughout the remelted layer. In a word, the LR process is beneficial to the reduction of segregation phase. The thick and long strip-like Laves phase completely disappeared and was replaced by the refined and dispersive ones in the remelted region.

### 3.3. Hardness

**Figure 9:**
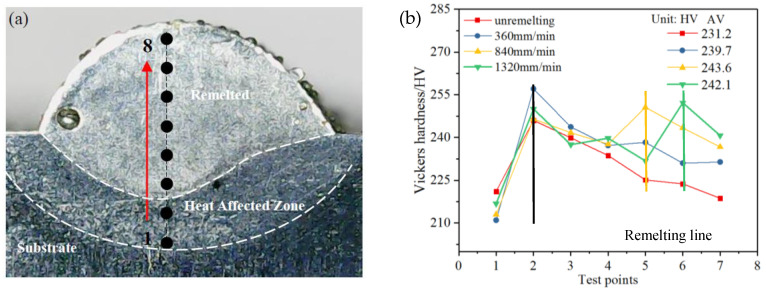
(**a**) The test points of the hardness on the cross section of the single-pass layers; (**b**) Vickers hardness results of the test points.

Three groups of single-pass remelted layers ($v_{\mathrm{LR}}=$ 360, 840 and 1320 mm/min) were selected for comparison with the LMD layers in hardness. Figure 9 reveals the micro-hardness distribution at a cross-section of the LMD + LR layer along the depth direction (red line in Figure 9a). The distance between adjacent test points is 400 μm. For each test point, the reported Vickers hardness was determined by the average of at least three times measurements. During the LMD process, the substrate underwent the tempering process, leading to the creation of the Heat Affected Zone (HAZ). In Figure 9a, the HAZ is marked in the white dotted line and the lowest hardness, 211.5 HV appears on the HAZ. In Figure 9b, compared with the LMD layers, the average hardness (AV) of the remelted layers increases significantly. The average hardness of LMD + LR layers gradually decreases from the bottom to the top of the layer, which is consistent with that of LMD layers. However, when $v_{\mathrm{LR}}>$ 360 mm/min, the cladding layers were incompletely melted and the hardness (yellow and green lines in Figure 9b) above the remelting line increases obviously. As $v_{\mathrm{LR}}$ decreases, the hardness of the remelted zone increases gradually. In addition, Figure 9b also proves that the scanning speed has a little effect on the average hardness and the maximum AV difference is only 12.4 HV, 5.12% of the maximum AV ($v_{\mathrm{LR}}=$ 1320 mm/min).

Based on the above experimental results, two main factors contribute to the hardness variations: Laves phase and pores. The creation of Laves phase plays a negative influence on the mechanical properties of Inconel 718 parts formed by LMD process. On one hand, excessive Laves phase in the cladding layer will consume the strengthening alloying elements such as Mo and Nb, which obstructs the formation of the strengthening phase. On the other hand, existence of pores reduces the inner cohesion of cladding layer and makes it easy to deform under external force. According to the above results, it can be deduced that the content of Laves phase and the porosity in the cladding layer will be reduced after LR treatment. The low content of Laves phase and porosity also contribute to the growth of average hardness for the LMD + LR layers.

Moreover, a high laser scanning speed in the LR process is in favored of refining the Laves phase. The strengthening alloying elements, Nb and Mo tend to be deposited in the gray matrix. However, excessive remelting scanning speed ($v_{\mathrm{LR}}=$ 1320 mm/min) also increases porosity, which can lower the hardness. Therefore, considering the comprehensive influence of Laves phase and porosity, the average hardness of both LMD and LMD + LR layers has no obvious difference under different laser scanning speeds.

## 4. Conclusions

The effect of laser remelting on cladding layer of Inconel 718 superalloy formed by laser metal deposition was studied in this paper. Different laser scanning speeds can obviously change the microstructural evolutions, Laves phase and hardness of the cladding layers. The LR process can promote the formation of columnar dendrites. Columnar dendrites uninterruptedly grow from the bottom to the top under the low laser scanning speed. The higher laser scanning speed has a significant effect on refining Laves phase and reducing Nb segregation. When the laser scanning speed of the LR process is equal to that of LMD (360 mm/min), the cladding layers can be completely remelted and the content of Laves phase per unit area of the LMD + LR layer is 22.4% lower than that of the LMD layer. As the laser scanning speed increases from 360 to 1320 mm/min, the mean primary dendrite arm spacing (PDAS) values of the remelting area decrease from 6.35 μm to 3.28 μm gradually. In addition, the LR process is beneficial to the reduction of segregation phase. The thick and long strip-like Laves phase completely disappeared and was replaced by the refined and dispersive ones in the remelting region. The LR treatment can also slightly improve the hardness of single-pass cladding layers. The low content of Laves phase and porosity also contribute to the growth of average hardness for the LMD + LR layers. However, the laser scanning speed has a slight effect on the average hardness of both LMD and LMD + LR specimens and the maximum average hardness difference is only 12.4 HV.

## Figures and Tables

**Table 1:** Chemical composition of nickel-based superalloy Inconel 718 powder.

Element	Ni	Cr	Mo	Nb	C	Al	Ti	Mn	Cu	Fe
wt.%	balanced	18.165	3.228	5.395	0.064	0.625	0.928	0.158	0.134	20.886

**Table 2:** Element content analysis of different precipitates.

Element	P1	P2	P3	P4
	wt.%	wt.%	wt.%	wt.%
C	8.00	6.82	9.07	6.98
Ti	11.58	0.69	17.97	1.46
Cr	12.17	18.65	10.83	12.85
Fe	11.16	18.83	10.84	11.38
Ni	31.30	49.93	32.92	35.39
Nb	19.04	2.66	14.52	21.94
Mo	7.75	2.96	3.85	10.00
